# Reproducibility of 3-Dimensional Ultrasound Measurements of Placental Volume at Gestational Ages 11 – 14 Weeks

**Published:** 2015-12-28

**Authors:** ML Larsen, KV Naver, MM Kjaer, FS Jorgensen, L Nilas

**Affiliations:** Department of Obstetrics and Gynaecology, University Hospital Hvidovre, University of Copenhagen, Denmark.

**Keywords:** Placental volume, variability, reproducibility, three-dimensional ultrasound, virtual Organ Computeraided AnaLysis, VOCAL™

## Abstract

**Objective:**

To evaluate the reproducibility of placental volume using three-dimensional ultrasound.

**Methods:**

The VOCAL (Virtual Organ Computer-aided AnaLysis) technique involves rotating an image of an object along an established axis using predefined angles. This provides a number of sections to measure manually, resulting in the object being displayed with an estimated placental volume. Four predefined angles 30°, 15°, 9°, and 6°, creating 6, 12, 20 and 30 sections, respectively. Measurements of placenta volumes in 21 women with singleton pregnancies were performed at gestational age 11-14 weeks by a single consultant in Foetal Medicine and later processed by two observers. The intraobserver reproducibility between all four angles was calculated as the mean Coefficient of Variation. Interobserver reproducibility was assessed by Interclass Correlation Coefficient (ICC), Limits of Agreement (LOA) and illustrated in Bland-Altman plots.

**Results:**

There was no significant difference in intraobserver variability between the four angles, p = 0.19, but a trend towards a lower coefficient of variation with the smallest angle was observed. A high intraobserver reproducibility was found using the 6° angle (ICC = 0.918 (0.812 - 0.966) and 0.983 (0.960- 0.993), LOA = [−22.9- 22.5] and [−14.3 - 12.1]), but interobserver reproducibility showed a wide range of agreement (LOA = [−50.5- 34.8]), particularly in cases with u-shaped placentas.

**Conclusion:**

The low interobserver reproducibility of VOCAL measurements of placentae requires significant differences between normal and abnormal cases if the technique should be implemented for clinical use.

## Introduction

Placental dysfunction is a major cause of pregnancy complications such as foetal growth restriction, placental abruption, perinatal pregnancy loss and pregnancy-induced hypertension (Hata et al., 2011), which are all associated with high maternal and perinatal mortality and morbidity. Many studies suggest a relationship between placental size and adverse late-pregnancy outcomes (Godfrey et al., 1991; Ananth et al., 1999; Lao et al., 2000; Schuchter et al., 2001; Metzenbauer et al., 2002; Rizzo et al., 2008; Effendi et al., 2014). It may therefore be relevant to have a reliable technique that could measure the size of the placenta at the early stages of pregnancy and thereby identify pregnancies at risk of complications in the second or third trimester..

Three-dimensional (3D) ultrasound is a noninvasive but time-consuming measurement technique that may be useful in research on placental characteristics and placental function. The Virtual Organ Computer-aided AnaLysis (VOCAL) technique involves rotating an image of an object along an established axis using predefined angles. This provides a number of sections for manual measurements in order to estimate placental volume. Four predefined angles exist 30°, 15°, 9°, and 6°, creating 6, 12, 20 and 30 sections, respectively. The VOCAL technique has been found to be equivalent and to some extent superior, compared to other 2D and 3D ultrasound measuring techniques in both usage of time and precision (Raine-Fenning et al., 2003; Nowak et al., 2008; Cheong et al., 2010).

The present study had two aims. The first aim was to estimate the intraobserver variability for one observer using VOCAL to estimate placental volume in early pregnancy with all four different predefined angles of rotation. Our hypothesis was that using a smaller angle giving more sections to manually measure would present less variation.

The second aim was to estimate the interobserver reproducibility between two observers using the angle of rotation with the smallest intraobserver variation. Our hypothesis was that placenta volumes estimated with VOCAL are highly reproducible. This is to our knowledge the first in vivo study to evaluate the intraobserver reproducibility of four different angles of rotation and also the first study to evaluate the interobserver reproducibility for the 6° angle of rotation.

## Materials and Methods

This was a prospective observational clinical study at the Foetal Medicine Unit of the Department of Obstetrics and Gynaecology, at Copenhagen University Hospital Hvidovre, during the time period 2010-2011 involving 21 nulliparous women with live singleton pregnancies between gestational age 11 + 4 to 14 + 6 weeks of gestation, and all with BMI < 35. The patients were recruited from a study on Polycystic Ovary Syndrome (PCOS) and pregnancy (Aziz et al., 2012), and included women with and without PCOS. All had a normal first trimester combined screening including nuchal translucency prior to the 3D ultrasound examination.

A consultant in Foetal Medicine scanned all 21 patients trans abdominally with a Voluson E8 Expert US machine. Additionally, one observer confirmed inclusion of the entire placenta.

The volumetric estimations of each of the 21 placentas were performed twice in random order by a single blinded observer with each of the four different predefined angles of rotation: 30°, 15°, 9° and 6°, each providing 6, 12, 20 and 30 sections, respectively. The basal and chorionic plates delineating the placental and uterine wall were carefully excluded. In total 168 (21 × 2 × 4) placental volumes were defined manually. To investigate the interobserver reproducibility, a second blinded observer randomly estimated each placental volume twice, but only using the angle of rotation (6°) with the lowest variation, providing 42 (21 × 2) placental volumes.

Placental location and shape were not criteria for eligibility. Women with both anterior and posterior located and regular and u-shaped placentas (involving both the anterior and posterior wall) were included in the study.

The study was approved by the local Ethics Committee and the Danish Data Protection Agency (H-2-2010-022 and J. nr. 2011-41-6645). All the women who participated in the study received informed consent.

### Calculation and statistics

Systemic bias was determined by calculating the 95% confidence interval (CI) for the mean difference, finding zero within these values meaning no systematic bias. All measurements were confirmed as being normally distributed with the Shapiro-Wilk test.

Intraobserver reproducibility of the four predefined angles was calculated and expressed in percentages as a mean coefficient of variation. The difference between the placental volumes and coefficients of variation was calculated using a Bootstrap Permutation Test controlling for repeated depending measurements.

Intra- and interobserver reproducibility for the 6° angle of rotation was estimated using Intra- and Interclass Correlation Coefficients (ICC) and the 95% limits of agreement. Intraclass Correlation Coefficient was calculated using absolute agreement one-way ICC and Interclass Correlation Coefficient using two-way mixed ICC. Bland-Altman plots were constructed to present the agreement between the measurements. The plots present the difference in the measurements against the mean volume, with a 95% CI and limits of agreement. For calculation of interobserver reproducibility the mean volume of the measurements acquired by the first observer was compared with the mean volume of the measurements acquired by the second observer.

Statistical analysis was performed using the software SPSS for Mac, version 22.0 (SPSS, Chicago, IL, USA).

## Results

Median maternal age was 30 years (range 22-39 years), median gestational age was 13 + 5 weeks (range 11 + 4 to 14 + 6 weeks). Mean placental volume was 97.4 cm3 (SD ± 27.4) with a minimum of 51.9 cm3 and a maximum of 135.7 cm3 obtained by the first observer.

There was no statistically significant difference in using the four different angles of rotation (p = 0.19, Bootstrap Permutation Test), but there was a trend towards lower coefficient of variation with lower angle and more sections to define manually. The lowest coefficient of variation was 5.6% (± 4.7) for the 6° angle (Table I).

**Table I:** Intraobserver variation when measuring placental volume using the VOCAL technique with four different predefined angles of rotation, a total of 21 placentas were measured twice by a single observer at each angle.

Angle of rotation	Number of sections	Mean coefficient of variation (± SD)
6°	30	5.6% (± 4.7)
9°	20	6.0% (± 4.6)
15°	12	6.8% (± 4.1)
30°	6	8.6% (± 6.4)

* SD, standard deviation.

**Figure 1:**
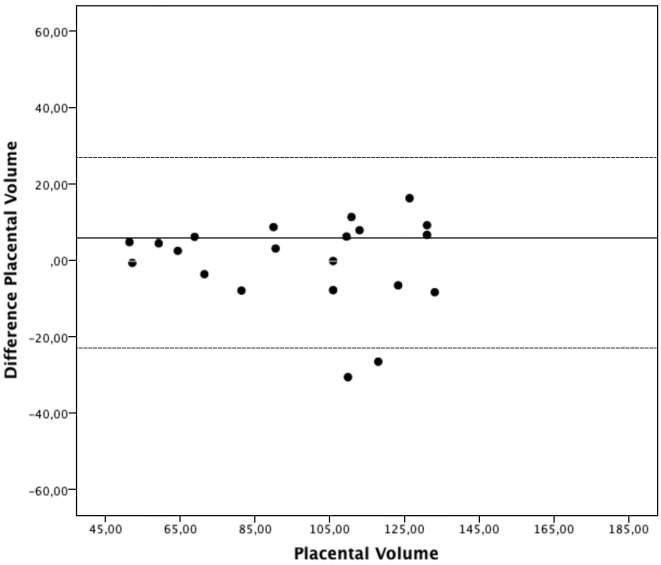
— Intraobserver agreement, observer 1. Bland-Altman plot presenting intraobserver agreement for the first observer. The differences between the measurements plotted against the mean placental volumes.

**Figure 2:**
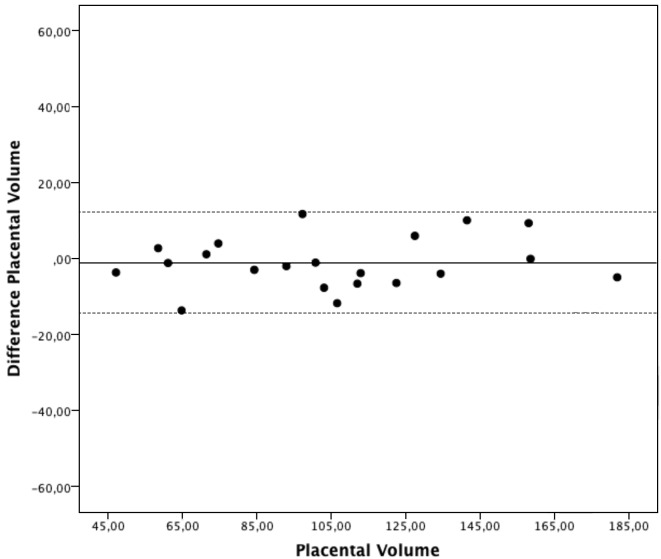
— Intraobserver agreement, observer 2. Bland-Altman plot presenting intraobserver agreement for the second observer. The differences between the measurements plotted against the mean placental volumes.

**Figure 3:**
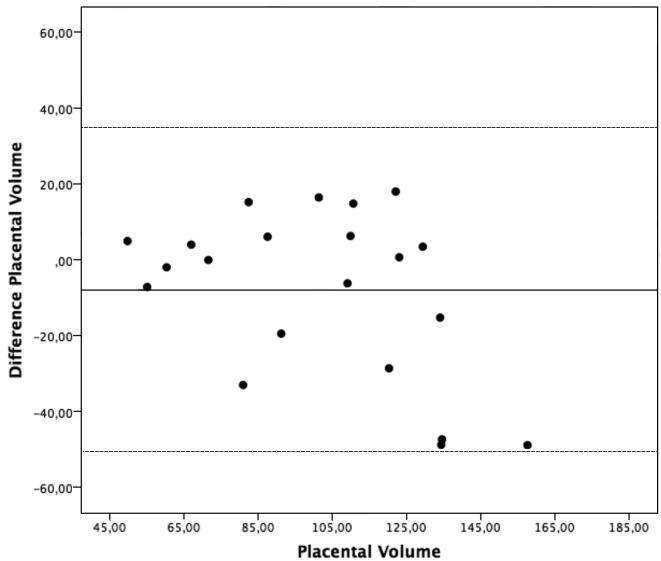
— Interobserver agreement. Bland-Altman plot presenting interobserver agreement between the two observers. The differences between the mean measurements plotted against the mean placental volumes.

The estimated intraobserver ICC and limits of agreement were 0.918 (0.812 - 0.966) and [−22.9 -22.5] for the first observer, and 0.983 (0.960 - 0.993) and [−14.3 - 12.1] for the second observer (Table II). The estimated interobserver ICC was 0.813 (0.680 - 0.909) and limits of agreement were [−50.5- 34.8] (Table II). Bland Altman plots for the intra- and interobserver agreements are shown in Figures 1, 2 & 3. No systematic bias was found. There was no clear relationship between the differences and the magnitude of the measurements. Log-transformed measurements did not improve the Bland-Altman plots.

**Table II:** Intra- and interobserver reproducibility when measuring placental volume using the VOCAL technique with the 6° angle of rotation, a total of 21 placentas were measured twice by each observer.

	Intraobserver reproducibility		
	Observer 1	Observer 2	Interobserver reproducibility
		
Mean difference	-0.2	-1.1	-7.8
95% CI	-5.5; 5.0	-4.2; 2.0	-17.8; 2.1
LOA	-22.9 - 22.5	-14.3 - 12.1	-50.5 - 34.8
ICC (95% CI)	0.918 (0.812 - 0.966)	0.983 (0.960 - 0.993)	0.813 (0.680 - 0.909)

* SD, standard deviation.

Of the 21 placentas, 12 (57%) were situated either at the anterior or posterior uterine wall and 9 (43%) were u-shaped. It was possible to obtain all placental volumes despite the position in the uterus. The mean difference and limits of agreement between the measurements of the two observers went from 4.3 cm3 and [−20.9 - 29.5] on the more consistently shaped placentas, to −23.9 cm3 and [−65.2 -17.2] on the u-shaped placentas.

## Discussion

This is to our knowledge the first in vivo study to evaluate the intraobserver reproducibility using VOCAL for first trimester placenta volume measurements with the four different angles of rotation. It is also the first study to evaluate the interobserver reproducibility on the 6° angle of rotation with 30 manually defined sections. We observed very good intraobserver reproducibility and interobserver reliability, but the interobserver agreement showed wide limits of agreement. When differentiating between location and shape of the placentas we found a much better interobserver agreement in placentas located either on the anterior or posterior uterine wall, while the interobserver agreement in u-shaped placentas were noticeably wider.

There was no statistically significant difference in the variation between the four angles of rotation (p = 0.19, Bootstrap Permutation Test), but there was a trend towards less variation with smaller angle and more sections, as expected in our hypothesis. This trend was the reason for choosing the 6° angle for further evaluations of VOCAL.

Another in vitro study has found the 6° angle to be more reliable than the other angles of rotation (Raine-Fenning et al., 2003). Only two in vivo studies have compared the reproducibility of more than one angle of rotation. In placentas at gestational age 11 to 14 weeks Cheong et al. (2010) found the best reproducibility in the lowest angles, while Nowak et al., (2008) at gestational age 7 to 10 weeks found the lowest angle to be equivalent with other angles of rotation. This disparity may be explained by the different gestational ages (Hata et al., 2011).

When using ICC a value between 0.61 to 0.80 implies good correlation and between 0.81 to 1.00 very good correlation (Jones et al., 2011). Our results show very good reliability using the 6° angle of rotation both within and between our two observers (Intra ICC: 0.918 (0.812- 0.966) and 0.983 (0.960- 0.993), Inter ICC: 0.813 (0.680 - 0.909)).

Therefore we consider VOCAL a reliable technique to estimate the size of the placenta with the size of measurement errors being small compared to the variation in the subjects. We also found good agreement within the two observers with limits of agreement between [−22.9-22.5] for the first observer and [−14.3-12.1] for the second observer. However the agreement between the two observers was wide [−50.5 - 34.8], which means we can expect the differences on 95% of future measurements between the two observers to lie between −50.5 and 34.8 cm3 (Bartlett and Frost, 2008). Other studies evaluating the reproducibility for the VOCAL technique to access placental volumes have obtained very good reliability and agreement (Mercé et al., 2004; De Paula et al., 2008; Cheong et al., 2010; Huster et al., 2010; Jones et al., 2011). Yet, most of them lack uniformity in their methodology and almost all of them use the 30° angle of rotation.

Factors disturbing measurements include interand intraobserver variation, intersubject variation, and interaction between the observer and the image. It was shown that a large part of the variation in placental measurements could be explained by differences in placental volumes (Hafner et al., 1998). However it has also been found that the shape of the object has more effect on reliability and validity than the size of the object (Raine-Fenning et al., 2003). With the placenta being very irregular in shape, the effect on measurement errors could be considered larger than for more consistently shaped objects. In our study the observers showed greater difficulty in agreeing on volumes of u-shaped placentas. More than 40% of placentas in our study were u-shaped and this is widely affecting the reproducibility. It is not possible to compare this with other studies since none of them include this factor.

Another aspect is difficulties in defining the placental boundary from the surrounding vascularity (Nelson et al., 2000; Chen et al., 2006; Deurloo et al., 2007; Jones et al., 2011). It is conceivable that with the 6° angle giving five times as many placental boundaries to define manually and five times as many sections for the observers to agree upon, compared with the 30° angle, this could widely affect the reproducibility.

Reproducibility is an umbrella term for reliability and agreement. Together it estimates the variation in measurements on the same subject made under changing conditions such as using a second observer or doing the measurements over a period of time (De Vet et al., 2006).

Only using ICC can give a flawed impression of the reproducibility (Bland and Altman, 1995) because low ICC values do not necessarily mean a reduction in the reliability of a test since ICC reflects both the measurement errors and the heterogeneity of the patients being examined. A decrease in the heterogeneity of the patients will result in lower ICC despite the size of the measurement errors remaining unchanged. This can produce falsely reassuring results (De Vet et al., 2006). Therefore, the limits of agreement and Bland Altman plots are included since they do not consider the difference between patients and only reflect how close the measurements are to each other (Bland and Altman, 1986). It is always important to remember that no statistical test can absolutely prove whether a test is reliable or not, as this is correlated to clinical judgement.

The limitations of this study are that we only focused on the reproducibility on assessing the volumes and did not include the ultrasound acquisition. A larger sample size would have strengthened our findings. Our population was included between gestational weeks 11 to 15 where the placenta is relatively small, but we did experience difficulties in including the whole placenta in the scan on women after week 14 with larger placentas. The reproducibility might have been better if the measurements had been performed earlier in pregnancy such as week 7 or 8 when the placentas were more regular in shape and with smaller volumes. On the other hand, the differences between placental volume in pregnancies with and without complications might be less pronounced in early gestation. The optimal time for assessing placental volume has not been systematically investigated.

The wide limits of agreement between the two observers implies that if placental volume is to be used as a pathological indicator, the real difference between sick and healthy placentas has to be somewhat large if physicians are to use the VOCAL technique with 6° angle of rotation at gestational week 11 to 14. The method does not seem sensitive enough to detect smaller differences in size particularly when measuring irregular shaped placentas, questioning the clinical relevance of using placental volume measured in early pregnancy as an estimator of the risk in pregnancy outcome. Further prospective studies are required to identify if VOCAL is sensitive and specific enough to identify and differentiate early placental dysfunction.
